# Targeted massively parallel sequencing for congenital generalized lipodystrophy

**DOI:** 10.20945/2359-3997000000278

**Published:** 2020-08-24

**Authors:** Aline D. Costa-Riquetto, Lucas S. Santana, Lílian A. Caetano, Antônio M. Lerário, Joya E. M. Correia-Deur, Débora R. Bertola, Chong A. Kim, Márcia Nery, Alexander A. L. Jorge, Milena G. Teles

**Affiliations:** 1 Universidade de São Paulo Faculdade de Medicina Hospital das Clínicas São Paulo SP Brasil Grupo de Diabetes Monogênico, Unidade de Endocrinologia Genética/Laboratório de Investigação Médica (LIM/25) e Unidade de Diabetes, Hospital das Clínicas, Faculdade de Medicina, Universidade de São Paulo, São Paulo, SP, Brasil; 2 Universidade de São Paulo Faculdade de Medicina Hospital das Clínicas São Paulo SP Brasil Unidade de Endocrinologia Genética, Laboratório de Endocrinologia Celular e Molecular/Laboratório de Investigação Médica (LIM/25), Hospital das Clínicas, Faculdade de Medicina, Universidade de São Paulo, São Paulo, SP, Brasil; Departamento de Medicina Interna, Divisão de Metabolismo, Endocrinologia e Diabetes, Universidade de Michigan, Ann Arbor, MI, USA; Universidade de Michigan Divisão de Metabolismo, Endocrinologia e Diabetes Departamento de Medicina Interna Ann Arbor MI USA; 3 Universidade de São Paulo Faculdade de Medicina Hospital das Clínicas São Paulo SP Brasil Unidade de Endocrinologia Genética, Laboratório de Endocrinologia Celular e Molecular/Laboratório de Investigação Médica (LIM25), Hospital das Clínicas, Faculdade de Medicina, Universidade de São Paulo, São Paulo, SP, Brasil; 4 Universidade de São Paulo Instituto da Criança Unidade de Genética São Paulo SP Brasil Unidade de Genética, Instituto da Criança, Universidade de São Paulo, São Paulo, SP, Brasil; 5 Universidade de São Paulo Faculdade de Medicina Hospital das Clínicas São Paulo SP Brasil Unidade de Diabetes, Hospital das Clínicas, Faculdade de Medicina, Universidade de São Paulo, São Paulo, SP, Brasil

**Keywords:** Congenital generalized lipodystrophy, Berardinelli-Seip syndrome, massively parallel sequencing, deep sequencing

## Abstract

**Objective:**

Our aim is to establish genetic diagnosis of congenital generalized lipodystrophy (CGL) using targeted massively parallel sequencing (MPS), also known as next-generation sequencing (NGS).

**Subjects and methods:**

Nine unrelated individuals with a clinical diagnosis of CGL were recruited. We used a customized panel to capture genes related to genetic lipodystrophies. DNA libraries were generated, sequenced using the Illumina MiSeq, and bioinformatics analysis was performed.

**Results:**

An accurate genetic diagnosis was stated for all nine patients. Four had pathogenic variants in *AGPAT2* and three in *BSCL2*. Three large homozygous deletions in *AGPAT2* were identified by copy-number variant analysis.

**Conclusions:**

Although we have found allelic variants in only 2 genes related to CGL, the panel was able to identify different variants including deletions that would have been missed by Sanger sequencing. We believe that MPS is a valuable tool for the genetic diagnosis of multi-genes related diseases, including CGL.

## INTRODUCTION

Congenital generalized lipodystrophy (CGL) or Berardinelli-Seip syndrome is a rare group of autosomal recessive diseases characterized by the near-complete loss of adipose tissue (1,2). The loss of body fat occurs at birth or within the first year of life, and is associated with prominent muscles and hepatosplenomegaly (3). Because of a leptin deficiency, patients with CGL develop hyperphagia, hyperinsulinemia, and hypertriglyceridemia at an early age. Later, usually in adolescence or early adulthood, affected individuals may present with diabetes mellitus (DM) and severe hepatic steatosis (2,4). There are four genetic subtypes of CGL (CGL1-CGL4) caused by mutations in *AGPAT2* (OMIM #608594), *BSCL2* (OMIM #269700), *CAV1* (OMIM #612526), and *PTRF* (OMIM #613327), respectively (5). The majority of the approximately 300 to 500 reported cases of CGL worldwide are caused by mutations in either *AGPAT2* or *BSCL2* (6). Genetic diagnosis enables counseling of affected families and determination of the optimal therapeutic strategy, such as administration of recombinant human methionyl leptin (6,7). Definitive diagnosis of CGL is usually based on genotyping using Sanger sequencing (8).

However, Sanger sequencing is laborious and time-consuming, specifically for large genes and diseases caused by multiple candidate genes (9). In addition, Sanger technique is not suitable for the detection of large deletions (9). Recently, massively parallel sequencing (MPS), also known as next-generation sequencing (NGS), has been demonstrated to be a cost-effective alternative for the diagnosis of monogenic diseases, overcoming the limitations of Sanger sequencing and enabling the simultaneous analysis of different genes and patients (10). In this study, we performed targeted MPS using a customized panel to demonstrate its application in the genetic diagnosis of patients with CGL.

## SUBJECTS AND METHODS

### Patients and ethical statement

We studied nine probands. They were unrelated, one male and 8 females with a clinical diagnosis of CGL, which was determined using the American Association of Clinical Endocrinologists consensus statement for the detection of lipodystrophy (2). Our probands were identified as Proband 1 (P1) to Proband 9 (P9), and were followed at Diabetes Outpatient Clinic of the University of São Paulo Medical School. The median age of probands was 15 years old (1.4 to 38.8). Of note, all patients presented a generalized lack of adipose tissue in the first year of life and muscular hypertrophy. At physical examination, P 5-7 had absence of fat even in palms and soles, and that was preserved in P 1-4 and P9. Six of the nine (6/9) participants were from consanguineous families. Information regarding consanguinity for P2 was not available as the patient was adopted. Six of the nine (6/9) probands presented DM with median age of onset of 15 years old (4 to 18). Four out of the six patients (4/6) with DM were using high insulin doses. Almost all (8/9) had hypertriglyceridemia. Only three of them (3/9) did not present hepatomegaly and four (4/9) had hepatic steatosis. Three of the patients (3/9) had cardiomiopathy. The clinical features of all probands are summarized in Table 1. The Institute's Ethics Committee approved the study and the patients and/or legal guardians gave written informed consent.

**Table 1:** Clinical features and genotype of patients with CGL

Proband^1^	P1	P2	P3	P4	P5	P6	P7	P8	P9
Gene	*AGPAT2* 2	*AGPAT2* 2	*AGPAT2* 2	*AGPAT2* 2	*BSCL2* 3	*BSCL2* 3	*BSCL2* 3	*AGPAT2* 2	*BSCL2* 3
Nucleotide change	c.646A>T	c.366_588+534del	c.589-2A>G	c.366_588+534del	c.412C>T	c.192_193delCCinsGGA	c.325dupA^4^	c.366_588+ 534del	**c.222_223del**+ c.213-11A>G
Protein change	p.Lys216*	p.Gly106fs*188	p.Gln196fs*228	p.Gly106fs*188	p.Arg138*	p.Ser64Argfs*12	p.Thr109Asnfs*5	p.Gly106fs*188	**p.Cys74fs** + g.7286A>G
Consanguinity	+	NA	+	+	+	+	+	−	−
Current age (years)	23.2	4.5	26.5	38.8	15.0	34.3	5.0	17	1.4
Gender	F	F	F	F	F	F	F	M	F
TG (mmol/L)^5^	133.5	4.8	40.9	22.1	11.1	34.1	28.2	3.4	60.3
TC (mmol/L)	29.9	4.2	16.7	6.9	4.9	11.5	6.2	6.8	9.5
HDL (mmol/L)	2.4	0.5	1.4	0.6	0.7	1.1	NA	2.5	0.45
Leptin (μg/L)	< 0.5	0.9	< 0.5	NA	< 0.5	NA	NA	NA	NA
Hepatic steatosis	+	−	−	+	−	+	−	−	+
Hepatomegaly	+	−	+	+	+	+	−	−	+
Echocardiogram	Normal	Normal	Normal	CHLV	CM	CHLV + SH	Normal	NA	Normal
DM		−	+	+	+	+	−	+	−
DM age of onset (years)	15	NApl	13	15	4	18	Napl	NA	NApl
TDDI (IU/kg/day)	2.1	NApl	2.8	6.8	Not performed	2.0	Napl	0.55	NApl

Abbreviations: +: present; -: absent; CGL: congenital generalized lipodystrophy; CM: cardiomyopathy; CHLV: concentric hypertrophy of left ventricle; DM: diabetes mellitus; F: female; HDL: high-density lipoprotein; IU: international unit; NA: not available; NApl: not applicable; SH: septal hypertrophy; TC: total cholesterol; TDDI: total daily dose of insulin; TG: triglycerides.

1 Probands are identified as P1-P7.

Reference sequences:

2 NM_006412 and

3 NM_032667.6.

4 Formerly described as 669insA (NM_001122955.3).

5 Maximum value.

Bold: variant not described previously.

### Targeted array design

We designed a customized panel to capture the coding regions and intron-exon boundaries of the following genes related to CGL: *AGPAT2, BSCL2*, *CAV1* and *PTRF. LMNA* and *ZMPSTE24* were also included, as differential diagnosis of congenital generalized lipodystrophies, as well as familial partial lipodystrophy genes. The length of target fragments was approximately 488 kb, which spans all the exons examined.

### Sample preparation and sequencing

DNA was extracted from peripheral blood leucocytes using standard in-house protocols based on a salting-out method (11). DNA libraries were prepared according to the manufacturer's instructions using the SureSelect XT Library Prep Kit ILM (Agilent Technologies, Santa Clara, CA, USA). Extracted DNA quality and yield was measured using a Tape Station (Agilent Technologies). Enriched target regions were sequenced (2 × 150 bp paired-end reads) using a MiSeq Analyzer (Illumina, San Diego, CA, USA).

### Data analysis

Bioinformatics analysis was performed at the Laboratório de Sequenciamento em Larga Escala da FMUSP (SELA) using in-house analysis pipelines. Briefly, following quality control, reads were aligned to the reference human assembly (version b37 GRC/NCBI) using the Burrows-Wheeler Alignment Tool (BWA-MEM) (12). The resulting alignments were sorted by coordinates using Bamsort from the Biobambam2 suite (13). FreeBayes was used to call single nucleotide variants (SNVs) and small insertions and deletions (indels) (14), which were annotated using ANNOVAR (15). Copy-number variation (CNV) analysis was performed using Copy Number Targeted Resequencing Analysis (CONTRA) (16). Metric analyses of the raw reads and alignments were performed using FASTQC and QUALIMAP, respectively (17,18). Pathogenicity of identified variants were classified according to guidelines from the American College of Medical Genetics and Genomics and the Association for Molecular Pathology (ACMG/AMP) (19).

## RESULTS

**Figure 1:**
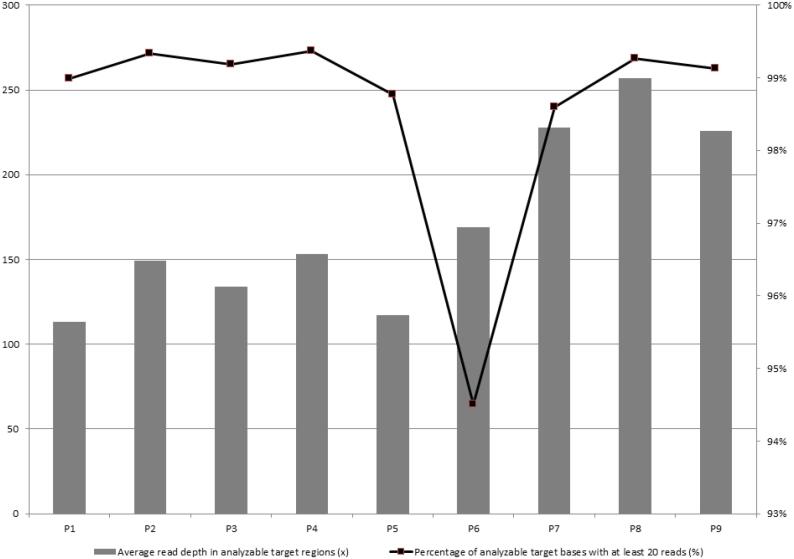
Sequencing performance of customized targeted massively parallel sequencing panel for congenital generalized lipodystrophy (CGL). The sequence depth of analyzable target regions (Y-axis on the right) and the percentage of analyzable target bases with at least 20 reads (Y-axis on the left) are provided for each proband (P1-P7) shown on the X-axis.

The run produced an average of read depth in analyzable target regions of 172 ± 49 per base with a median of 153 (range 113-226). Overall, > 98% of target regions had at least 20× coverage. Sequencing performance statistics are shown in detail in Figure 1 and Table 2.

**Table 2:** Performance statistics of targeted massively parallel sequencing using a customized CGL gene panel

Target region capture statistics	Proband^1^								
	P1	P2	P3	P4	P5	P6	P7	P8	P9
Average read depth in analyzable target regions	113	134	117	169.5	149	153	228	257	226
Percentage of analyzable target bases with at least 10 reads (%)	99.5	99.6	99.3	98.2	99.6	99.7	99.4	99.8	99.8
Percentage of analyzable target bases with at least 20 reads (%)	99.0	99.2	98.8	94.5	99.3	99.4	98.6	99.27	99.13

Abbreviation: CGL: congenital generalized lipodystrophy.

1 Probands are identified as P1-P9.

We identified centens of coding variants in the 4 CGL genes in our nine probands. Of these variants, 22 were extremely rare, with an allele frequency less than 1% according to publicly available genomic databases (1000 Genomes Project and the Exome Aggregation Consortium – ExAC), of which seven were disease causing variants (20,21). Moreover, CONTRA enabled the identifications of three deletions.

**Figure 2:**
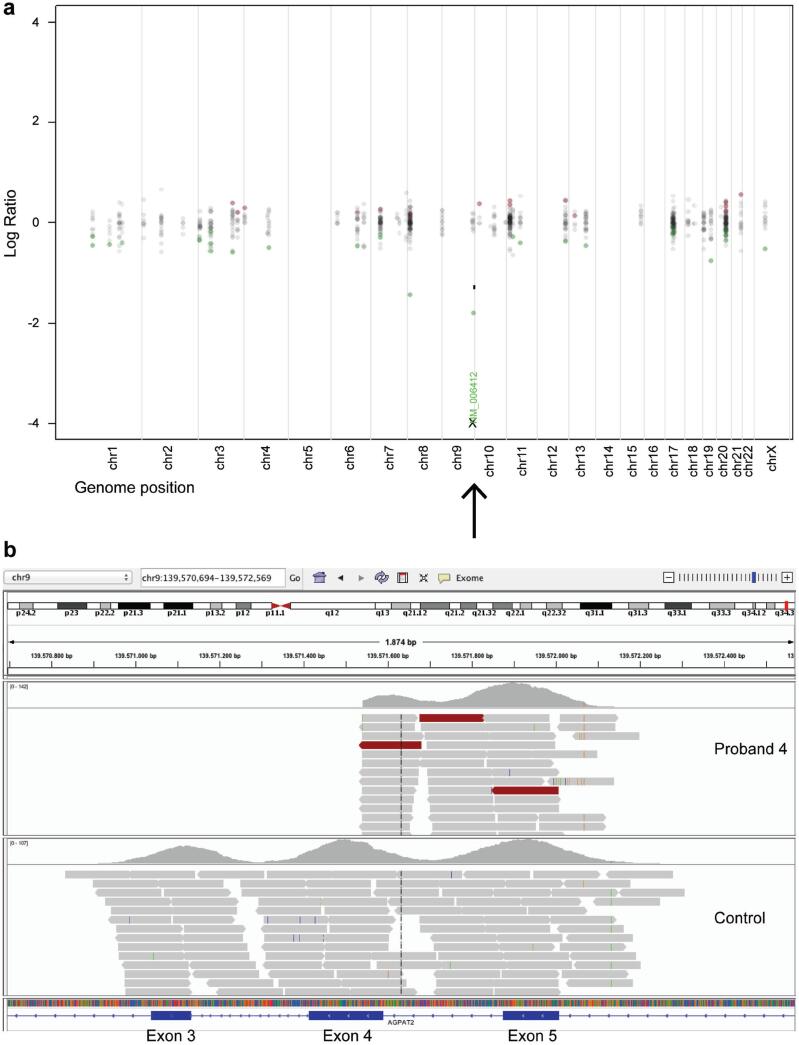
Deletion of exon 3 and part of exon 4 in *AGPAT2*. **A**) Copy Number Targeted Resequencing Analysis (CONTRA) plot of log ratio versus genome position showing deletion in *AGPAT2* (arrow). **B**) Integrative Genomics Viewer (IGV) image of *AGPAT2* in Proband 4 and a control.

**Figure 3:**
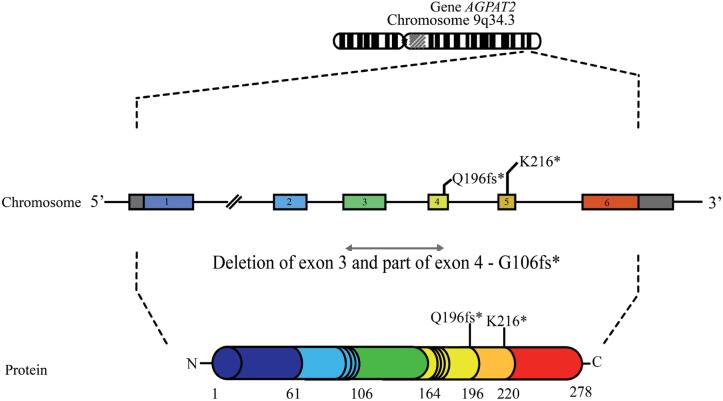
Schematic representation of the *AGPAT2* gene and its protein, with the location of the variants found in this study. Exons numbered from 1 to 6, with their respective coded regions represented with the same color.

Three of these variants were located in *AGPAT2* and resulted in a premature termination codon. P1 harbored a nonsense mutation in exon 5 (c.646A>T/p.Lys216*), whereas P3 had a splice-site mutation (c.589-2A>G) that produced a frameshift (p.Gln196fs*228). P2, P4 and P8 had the same large homozygous deletion of exon 3 and part of exon 4, that caused a frameshift mutation (p.Gly106fs*188) (Figure 2). Schematic representation of the *AGPAT2* gene and its protein, with the location of the variants found in this study are shown in Figure 3.

In four of our nine patients, we identified mutations in *BSCL2*, which resulted in a premature stop codon. P5 harbored a C-to-T transition (c.412C>T/p.Arg138*), P6 had a frameshift indel (c.192_193delCCinsGGA/p.Ser64Argfs*12), P7 had a frameshift duplication (c.325dupA/p.Thr109Asnfs*5) and P9 was compound heterozygote (c.222_223del/p.Cys74fs + c.213-11A>G/g.7286A>G).

**Figure 4:**
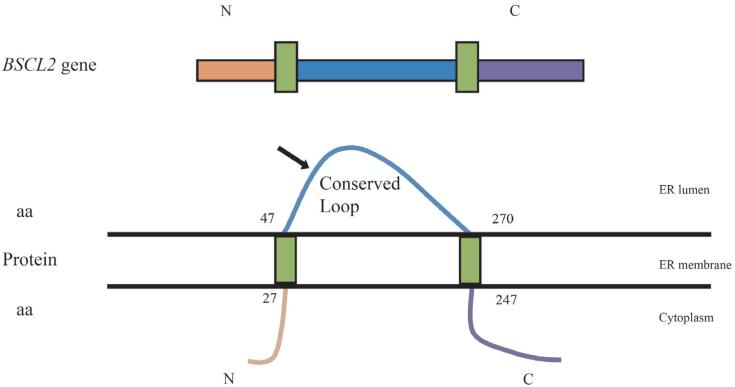
Schematic representation of *BSCL2* and seipine. The colors of the gene representation correspond to the portions encoded in the protein. In green, transmembrane domains. All variants found in this study are located in the conserved loop (*).

Only one found variant were not previously described (c.222_223del/p.Cys74fs). Schematic representation of *BSCL2* and seipine, as well the variants found in this study are shown in Figure 4.

All variants were considered pathogenic based on ACMG/AMP diagnostic criteria. They were confirmed using Sanger sequencing.

## DISCUSSION

Our custom-designed panel enabled the genetic diagnosis of all nine probands with CGL, which demonstrates targeted MPS is an accurate and efficient method for the genetic diagnosis of CGL. We had a robust sequencing run obtaining 98.4% of analyzable target bases with at least 20× reads. The 1.6% of analyzable target bases that did not achieve a minimal 20× read coverage either were from genes not associated with CGL or were located in untranslated regions. An exception was found in P4 that had poor coverage of exons 1, 2, and 11 in *BSCL2.* However, this proband had a pathogenic deletion of exons 3 and 4 in *AGPAT2*.

Although this is a study with only nine patients, we can recognize clinical similarities with those previously reported. Hypertriglyceridemia and hepatomegaly are, as described, prevalent in our probands. Besides, almost all patients with CGL-2 had absence of fat in palms and soles, in contrast to those with CGL-1. Interestingly, the only case of CGL-2 in which there was no fat loss in the palms and soles was of a 1.4-year-old female, leading us to question whether this loss of mechanical fat would not occur later in life. Patients with CGL have an increased incidence of cardiomyopathy, that is more frequent in CGL-2 (2,3,6,7). We found that two of our four patients with *BSCL2* mutations and only one of our patients with *AGPAT2* mutations presented with this condition. Forty-five percent of patients with lipodystrophy may develop DM, usually in the second decade of life (6). We found the majority of patients (6/9) with DM. Patients with CGL and DM usually require higher doses of insulin (>2 IU/Kg/d), as we can see in most (4/6) of our patients with diabetes. Metreleptin, recombinant human methionyl leptin, is the first-line treatment for endocrine comorbities and insulin therapy can be reduced and even discontinued with its introduction (7). Unfortunately, in our country, metreleptin is not yet approved by regulatory government agencies.

Although there are peculiarities in the phenotype when different CGL genes are affected, they can be very subtle to guide Sanger sequencing. Additionally, not all clinical features are present in the early manifestation of the condition (stage when is hard to distinguish the types of CGL), so it is important to have a wide genetic approach. MPS can promptly investigate a number of patients through a single testing run while simultaneously examining a large number of genes for each patient (9). Furthermore, because of cost reductions and workflow improvements, the widespread application of a common large-scale sequencing platform for multiple tests is a prevailing trend in genetic analysis (9,20).

In this study, CONTRA was relevant, as an effective CNV detection tool. It uses base-level log-ratios, which permits the inference of copy number gain and loss for each region, estimating a significance based on the null distribution of log-ratios (16). We found that using CONTRA was important to overcome this limitation of conventional algorithms, and consequently, enabled the identification of two large deletions in our cohort. These large deletions would not have been detected by conventional bioinformatics analysis of MPS, neither by Sanger sequencing.

On the other hand, we argue if MPS would be the most cost-effective approach instead of Sanger sequencing, since there are only two genes, *AGPAT2* and *BSCL2,* responsible for 90 to 95% of cases of CGL (21). Currently, 37 different variants in *AGPAT2* are reported in 126 subjects, found throughout the entire gene (22). The majority of variants are in intron 4 (c.589-2A>G/p.Gln196fs), described in 42 cases today, only one of them from Brazil. Twenty-seven percent of all variants in *AGPAT2* are within intronic sequences. Frameshift, nonsense and missense mutations are also frequent, in this order of prevalence (22). The type of variant does not seem to determine the phenotype or the severity of fat loss (6). In Brazil, studies have found that the homozygous deletion of exons 3 and 4 in *AGPAT2* are the most prevalent pathogenic variant in this gene (23–27). There are 18 cases described in our country that presents this deletion, mostly from the southern (22,24–27). We found three of our nine probands with this variant. Concerning to *BSCL2*, 36 variants have been described in 167 subjects. Most exons are affected by the variants described so far, with exon 4 being the most affected. Twenty-two percent are within intronic sequences, and nonsense mutations were more frequent, followed by frameshift and missense. The most prevalent is variant c.325dupA/p.Thr109Asnfs*5. In Brazil, it is in high prevalence in the northeast region, with 39 cases described (22,24,27). In our study, we found one of our nine probands with this variant (P7). Worldwide, the minority of the variants, both in *AGPAT2* and *BSCL2*, are homozygous (22). In Brazil, it is described only one compound heterozygous *AGPAT2* mutation and 2 cases in *BSCL2*. In our series we found only 1 case of heterozygosis composed in *BSCL2*, different from those described, one of which was not previously reported.

These findings show the importance of sequencing the two entire genes when studying lipodystrophy patients, that is more easily achievable with MPS.

Rehm et al., recommended that the first step for clinical application of MPS should be adoption of large multi-gene disease-targeted panels (9). A target panel for monogenic causes of diabetes, which includes genes that cause lipodystrophy, would be valuable in a diabetes research center as ours. In a referral center, the assemblage of all monogenic diabetes cases would justify MPS use.

In conclusion, our approach, which used a targeted MPS panel with standard bioinformatics analysis and CONTRA, enabled an accurate and rapid diagnosis in a small cohort of patients with CGL. It also allowed the identification of deletions that would not be able to be detected by Sanger sequencing. We identified seven different mutations in two of four genes associated with CGL. Our study demonstrated that targeted MPS is an efficient tool for the genetic diagnosis of CGL. Conversely, the advantage of routinely using MPS rather than Sanger sequencing out of the context of a diabetes center is still an issue to be further analyzed.
